# *‘A Different Ball Game’:* Adaptation of a men’s health program for implementation in rural Australia

**DOI:** 10.1186/s12889-023-16247-w

**Published:** 2023-07-19

**Authors:** Matthew D. McDonald, Kate Hunt, Joanna Moullin, Deborah Kerr, Nikos Ntoumanis, Eleanor Quested

**Affiliations:** 1 Curtin School of Population Health, Curtin University, Perth, Australia; 2 Physical Activity and Well-Being Research Group, enAble Institute, Curtin University, Perth, Australia; 3 Institute for Social Marketing and Health, Faculty of Health Sciences and Sport, University of Stirling, Stirling, UK; 4 Curtin Health Innovation Research Institute, Curtin University, Perth, Australia; 5 Danish Centre for Motivation and Behaviour Change, University of Southern Denmark, Odense, Denmark; 6 School of Health and Welfare, Halmstad University, Halmstad, Sweden

**Keywords:** Adaptation, Rural, Men’s health, Sport settings, Health behaviour, Inequalities

## Abstract

**Background:**

Men residing in rural areas are less likely to participate in weight management interventions than women, and few men-specific programs target rural areas. Aussie-Fans in Training (Aussie-FIT) is an evidence-based weight management intervention that uses professional Australian Football club affiliations and settings as a ‘hook’ to engage urban-residing men. The aim of this study is to report on how findings from rural stakeholder focus groups were used to inform the adaptation of Aussie-FIT for implementation in rural areas.

**Methods:**

Seven focus groups with stakeholders (*n* = 24) in three rural towns explored existing weight management and physical activity provisions, barriers and facilitators to engaging men, and considerations for adapting Aussie-FIT for implementation in rural contexts. Qualitative data were analysed using the framework approach. Adaptations made to the Aussie-FIT program and strategies to implement the program in rural contexts were reported using a structured framework.

**Results:**

Themes generated from our analysis include limited appealing services for men, Australian Football as a ‘common language’, the influence of the ‘smaller fishpond’(population), considerations for program inclusivity, and the importance of local partner organisations for sustainability. We adapted the recruitment and marketing strategies, delivery settings, football program theme and partnerships for rural implementation. Stakeholders advised that an Australian Football program theme without specific local club affiliations would be important to avoid alienating men with differing club allegiances or non-sporting backgrounds. A multi-component recruitment strategy utilising local trusted sources, and program marketing that aligns with masculine ideals were considered important by stakeholders in small communities where ‘people talk’.

**Conclusions:**

Rural areas were described as *‘a different ball game’* due to limited local services and resources in comparison to metropolitan areas. Study findings have synergies with previous studies undertaken in rural contexts including in relation to the power of word of mouth, the importance of trust, and local partner organisations. Findings have implications for engaging rural men in health interventions in rural contexts where professional sporting contexts are not available. Assessing the extent to which the adapted Aussie-FIT program can reach and engage men in rural Australia, and exploring the barriers and facilitators to delivering the program in rural contexts is required.

**Supplementary Information:**

The online version contains supplementary material available at 10.1186/s12889-023-16247-w.

## Introduction

Prevalence of obesity, cardiovascular disease, type 2 diabetes, and high blood pressure is higher in rural communities than urban areas [1–3]. Global rises in mean body mass index between 1985 and 2017 are more pronounced in rural than urban-residing men (2.1 vs 1.6 kg/m^2^) and women (2.1 vs 1.4 kg/m^2^) [4]. In Australia, obesity rates are highest in low socioeconomic rural communities, with obesity up to three times more prevalent (circa. 40% vs 13%) than affluent urban areas [5]. Increases in physical inactivity are more pronounced in rural areas [6], which is likely driven by technological advances increasing rural sedentary work time, particularly for men [7, 8]. In Australia and internationally, many rural communities face disadvantage due to a multitude of factors, including lack of access to facilities, resources, and services [2]. Despite a growing international evidence-base for rural weight management program effectiveness [9], rural men are significantly less likely to take up an offer to participate in mixed-gender programs than women [10]. The Australian Government’s Men’s Health Strategy highlights the need for interventions that support health in priority population groups, including men in rural and low socioeconomic areas [11]. However, men’s weight management programs seldom target rural or low socioeconomic areas [12].

One strategy that has proved valuable for engaging men internationally is to design group-based health programs specifically for delivery within sports settings in affiliation with professional clubs to appeal to their fanbases [13–17]. In the Aussie-Fans in Training (Aussie-FIT) pilot study, the program was delivered in affiliation with two Australian Football League (AFL) clubs in metropolitan Perth within professional club settings [18]. The AFL clubs promoted the program via their social media pages, and the program was highly attractive to urban-residing men [14]. The AFL club setting and the shared interest of participants in the AFL club was considered an important component that helped to attract men to the program and fostered within-group camaraderie [19]. In line with prior ‘Fans in Training’ programs [20], Aussie-FIT participants improved their mental and physical health, and reported positive health behaviour changes (e.g., diet and physical activity) [14]. However, Aussie-FIT is untested in rural areas and the program has not previously engaged a socioeconomically diverse sample of men [19]. Interventions that lack an evidence base to suggest they reach and are effective across demographic groups, may inadvertently exacerbate health inequalities [21, 22].

Australian Football is the most popular spectator sport in Australia [23], but professional AFL clubs are only located in major cities. Therefore, the original format of Aussie-FIT, which relies on program delivery in the AFL context, is unable to help address rural health inequities. To align with policy recommendations related to health inequalities [11, 24, 25], Aussie-FIT requires adaptation for implementation in rural contexts. Adaptation can be defined as the process of thoughtful and deliberate modification of intervention design or delivery, to improve intervention fit or effectiveness within a given context [26, 27]. Stirman et al.(2019) classify adaptations to evidence-based interventions into two broad categories: i) core intervention modifications; and ii) contextual modifications [28]. Context can be defined as a set of characteristics and conditions that alter, impede and/or support the delivery and effectiveness of interventions [29], and includes socioeconomic, geographical, and sociocultural contexts [30]. The degree of adaptation largely depends on differences or similarities between the new context and those from which evidence of effectiveness are derived [31]. In Aussie-FIT and other ‘Fans in Training’ programs, the ‘*behind the scenes*’ access and allure of professional sports club settings, including a stadium tour in session one, are typically emphasised in the program marketing materials [14, 19]. Without affiliation or access to professional club settings, adaptations to both the program content (e.g., the stadium tour) and implementation strategies (e.g., recruitment and marketing strategies) are required for Aussie-FIT to reach men in rural communities.

Recent guidance for adapting interventions highlights the importance of including a diverse range of stakeholders, including individuals and organisations that could facilitate intervention delivery or decisions about future intervention scaling [32]. The aim of this study is to report on how findings from rural stakeholder focus groups informed the adaptation of Aussie-FIT for implementation in rural areas. In doing so, we draw on concepts from implementation science and evidence on gender-tailored and sports setting-based interventions for men. The objectives of this study are to:

i)  explore the services available to support rural men to manage their weight or increase their physical activity
ii) examine barriers and facilitators to rural implementation and engagement of men across socioeconomic groups
iii) determine which specific adaptations are needed to implement Aussie-FIT in rural areas

## Methods

### Summary of Aussie-FIT

Aussie-FIT is a group-based gender-tailored behavioural weight management program for men (aged 35–65 with a BMI ≥ 28kg/m^2^) that has been tested in a pilot randomised controlled trial (RCT) in AFL settings in metropolitan Perth [14, 19]. The program consists of weekly AFL coach-led 90-min sessions over 12weeks that involves interactive education and physical activity components delivered in professional AFL contexts. Club coaches undertake 15-h of training with the research team. Funding is required to support the coaches training time, to cover 3h for each session delivered (1½ hours delivery, 1 ½ hours preparation), and for program resources (i.e., physical activity self-monitoring device and program booklet). The total direct costs (i.e., program set-up, promotion, and delivery costs) associated with the Aussie-FIT program in the metropolitan pilot was AUD$270 per participant [14].

Aussie-FIT capitalises on participants connection with their favourite AFL team, embeds behaviour change techniques (e.g., self-monitoring, goal setting and feedback on behaviours), and aims to foster a fun environment with positive humour [18]. The program content is informed by Self-Determination Theory and designed to empower men to use self-regulation strategies to support them to make positive changes to their physical activity and eating behaviours [18]. Further details can be accessed in the Aussie-FIT pilot RCT protocol [18], and a figure describing the key process evaluation functions is available in the process evaluation paper [19].

### Rural Aussie-FIT project

This study reports on the formative stages of a larger project that aims to help address the underrepresentation of men in rural and lower socioeconomic areas in community health programs. In this study, we engage local stakeholders in focus group discussions to inform the adaptation of Aussie-FIT for rural contexts. Building on this work, we continued to collaborate with the stakeholders that participated in this study and wider local networks to help support the implementation of the adapted Aussie-FIT program in rural towns. We plan to report on the implementation of Aussie-FIT in rural towns in a future publication using mixed-methods data, which will include implementation barriers and facilitators, and program reach, engagement, and retention.

### Setting

This study was undertaken in two ‘inner regional’ and one ‘outer regional’ towns in Western Australia, as classified by the Australian Bureau of Statistics [33]. The term ‘rural and remote’ encompasses all areas outside of Australia’s major cities, which includes ‘inner regional, ‘outer regional’, ‘remote’ and ‘very remote’ areas [34]. These sites were selected as they include areas with some of the highest obesity rates in Western Australia, higher levels of socioeconomic deprivation, and differing population sizes.

### Participants

Participants in the focus groups were staff or volunteers (aged > 18) working in rural areas in health promotion, men’s health, local football, sport development, community work, or similar roles.

### Recruitment

Staff from local partnership organisations (e.g., Department of Local Government, Sport, and Cultural Industries; Cancer Council; WA Country Health; WA Football Commission) supported researchers to identify and connect with potential contributors in the three planned rural Aussie-FIT delivery sites. Researchers also emailed various organisations (e.g., men’s sheds, local football clubs) to ask if they could share information about the Aussie-FIT focus groups to their members and networks.

### Stakeholder focus groups

Focus groups were used instead of individual interviews to meet the objectives of this study by capturing interactional discussions where participants could further develop or clarify their contributions, or introduce new viewpoints, with consideration of other participants’ perspectives. The focus groups were undertaken face-to-face at rural venues with a view to helping to build relationships between the researchers and the local stakeholders participating. Another pragmatic consideration was that the study researchers travelled to the rural locations in order to speak to participants face-to-face. Given the distance travelled, undertaking focus groups was considered the optimal way to allow the voices of the largest range of stakeholders to be heard (including those from diverse backgrounds) within the time available. Participants were given an information sheet, provided informed consent, and completed a short demographics form. Researchers introduced the Aussie-FIT program and pilot results using a 15–20min PowerPoint presentation. Researchers then used a topic guide (Appendix 1) to lead a discussion on local contextual considerations for the implementation of Aussie-FIT in rural settings. We developed the topic guide with a view to exploring local contextual factors, and to identify specific potential adaptations for rural settings (e.g., recruitment strategies, Australian football club theme, local sporting delivery settings). These topics were identified from previous research [18] and warranted further exploration in the present study due to differences between the metropolitan areas in which Aussie-FIT has previously been delivered and rural Australian contexts.

Twenty-four stakeholders participated across seven audio-recorded focus groups (mean length 57min, range 39–71min). Five focus groups were co-facilitated by two researchers (MMcD & EQ), and two by one researcher (MMcD). Participant characteristics are shown in Table 1. The overall sample was diverse with regard to occupation, work experience, age and gender, and representation of Aboriginal or Torres Strait Islander Peoples. Some of the individual focus groups were less diverse. For example, health promotion staff were the sole contributors to one group in site 1 and another in site 2, both of which lacked input from individuals in the sporting sector. Several participants had personal experience or interest in Australian football (e.g., former, or current player).

**Table 1:** Characteristics of focus group participants

Participant	Focus Group	Site^*^	Gender	Age	Ethnicity	Occupation Sector	Years’ Experience^*^
1	1	1	Female	30–39	White Australian	Health Promotion	6
2	1	1	Male	30–39	Indian	Health Programs	2.5
3	1	1	Female	30–39	White Australian	Health Promotion	1
4	2	1	Female	50–59	NR	Health Promotion	6
5	2	1	Male	18–29	White Australian	Allied Health	9
6	2	1	Male	18–29	White Australian	Men’s Health	1
7	2	1	Male	60–69	White Australian	Sport Sector	4
8	3	2	Male	50–59	White Australian	Sport Sector	30
9	3	2	Female	50–59	White Australian	Health Promotion	1
10	3	2	Female	40–49	White Australian	Sport Sector	4
11	3	2	Male	40–49	Aboriginal and/or Torres Strait Islander	Health Promotion	10
12	4	2	Female	40–49	White Australian	Health Promotion	3
13	4	2	Female	30–39	White British	Health Promotion	8
14	4	2	Female	30–39	White Australian	Student [Health Promotion]	n/a
15	4	2	Male	60–69	White Australian	Health Promotion	8
16	4	2	Male	20–29	Aboriginal and/or Torres Strait Islander	Health Promotion	1
17^#^	5 & 7	3	Female	18–29	White Australian	Health Promotion	3.5
18	5	3	Female	50–59	White Australian	Sport Sector	9
19	5	3	Male	NR	White Australian	Sport Sector	NR
20	6	3	Male	30–39	White British	Sport Sector	9
21	6	3	Female	18–29	White Australian	Sport Sector	4.5
22	7	3	Male	18–29	White Australian	Allied Health	7
23	7	3	Male	18–29	White Australian	Australian Football	4
24	7	3	Male	50–59	Aboriginal and/or Torres Strait Islander	Community Work	6

*NR* Not reported

^*^Sites 1 and 3 are classified as ‘inner regional’ and site 2 as ‘outer regional’ areas

^*^Years of experience in their current role or similar roles

^#^This individual participated in two focus groups

### Analysis

Anonymised verbatim focus group transcripts were read, reread, and entered into NVivo software to facilitate analysis guided by the framework approach [35]. This approach consisted of 5 steps: i) familiarisation; ii) thematic framework identification; iii) indexing; iv) charting; and v) mapping and interpretation [35]. The initial thematic framework construction was undertaken by A following data familiarisation. The framework was informed by emergent themes raised by participants as well as drawing upon a-priori issues deemed important by the researchers (e.g., potential recruitment strategies in rural locations). Another researcher (EQ) provided critical comment on the thematic framework based on detailed reading of three transcripts (one from each site). [MMcD] coded the interviews, charted context-specific factors by site and mapped key themes. [MMcD] and [EQ] met frequently to discuss the ongoing interpretation of the data. Differences in opinion were resolved through further discussion between [MMcD] and [EQ]. Driven by the goal of informing specific adaptations to the Aussie-FIT program for rural contexts, and to demonstrate exactly how these adaptations were derived, the analyses were intended to be at a more descriptive level. Themes are presented in a format that first reports findings related to the current context and local provisions to set the scene, before reporting on more specific themes that could inform future initiatives and Aussie-FIT adaptations.

Focus group findings were then used to inform adaptations to the Aussie-FIT program content and implementation strategies for rural Australian contexts. Adaptions to the program content for delivery in rural contexts were mapped against the original Aussie-FIT content delivered in urban professional AFL settings and reported using relevant questions from the Framework for Reporting Adaptations and Modifications (FRAME) [28]. Implementation strategy adaptations for rural contexts were similarly mapped against the original program and reported using relevant questions from the Framework for Reporting Adaptations and Modifications to Evidence-based Implementation Strategies (FRAME-IS) [36]. Using a structured framework provides a more systematic process to reporting adaptations, facilitates tracking and reflecting on how and why modifications have been made, and can allow for mechanisms of change conclusions to be drawn from the assessment.

## Results

This section presents the findings of the stakeholder focus group analysis, followed by a description of the specific adaptations made to the Aussie-FIT program for implementation in rural communities. Driven by the study objectives, five overarching themes were generated from the analysis. These were the ‘limited appeal of existing services to men’, ‘a common language’, ‘a smaller fishpond, ‘engaging rural men and diversity’ and ‘rural partnerships and sustainability’. The first theme most directly responds to the first study objective, to explore existing services in these rural communities. The second theme reports how Australian Football was considered a ‘common language’ in rural WA communities, with the sport described as extremely popular with strong rivalries between local clubs. The third describes the influence of the ‘smaller fishpond’ (population) within rural communities, including the importance of locally trusted community champions, and the potential benefits and drawbacks (‘double-edged sword’) linked to the power of word of mouth within close-knit communities. A variety of specific stakeholder recommendations around maximising the engagement of rural men including those from diverse backgrounds is then presented in the fourth theme. Finally, the importance of working closely with established organisations that are trusted, was considered vital for program sustainment. These findings directly informed the adaptations to Aussie-FIT for implementation in rural areas including to program recruitment and marketing strategies, the Australian Football program theme and linked program content, and planned partnerships with locally based organisations (see Table 2).

**Table 2:** Aussie-FIT program content and implementation strategy adaptations for rural Australian contexts guided by FRAME-IS and FRAME reporting frameworks

**Adaptation: Implementation Strategy, Type, Nature, and Level**	Pilot/Metropolitan Aussie-FIT	Rural Aussie-FIT	Rationale for Adaptation or Rural Specific Considerations	Exemplar Quotes and Linked Qualitative Themes Informing Adaptations	Generalisability, Fidelity, and Potential Outcome of Adaptation
Substitution of recruitment strategy content (organisational level)	Professional Australian Football League clubs affiliated with the Aussie-FIT programs shared promotional articles and recruitment information on their social media pages and websites to their large fan bases	Multi-faceted approach to recruitment including Facebook promotion, via local Australian Football clubs, local media coverage (radio and newspapers), word of mouth (e.g., via community champions, local organisations and other men interested in participating)	No affiliation with AFL clubs. The rural recruitment strategies were informed by stakeholder focus groups. The goal of this adaptation is to increase the reach and health equity relevance of the program	***Most Relevant Qualitative Themes and Sub-Themes:*** - A Smaller Fishpond - Trust, Recognition and Credibility: The Importance of Local Champions - A Double-Edged Sword ***Exemplar Stakeholder Quotations:*** *‘Hit the socials’[…]‘Site 1 community board goes off on Facebook’ (Site 1, FG2, Female)* *‘…leveraging the (local) clubs is definitely one way to do it’ (Site 1, FG1, Female)* *‘…bring a friend or two is probably going to have to be a realistic approach’ (Site 3, FG2, Male)* *‘It's probably the medium (newspapers) that works regionally, that doesn't work metro’ *(Site 3, FG2, Female) *‘…if you're talking about, how do we speak to the community, I guess I mean I, for our programs I use local radio. (FG1, Site 1, Female)*	This adaptation does not impact intervention delivery or likely effectiveness and is classified as fidelity consistent How effective the adapted recruitment strategy for rural areas is unknown and requires evaluation Given the smaller rural population sizes and the that well supported professional AFL clubs with large social media followings are not promoting the program, the rate of recruitment may be reduced in comparison to the metropolitan pilot ***Potential Generalisability:*** These recruitment approaches are potentially generalisable across rural Australian towns, for both the Aussie-FIT program and/or other health initiatives or target groups
Tailoring and adding/removing marketing strategy (implementer level)	Marketing of the program highlighted a *‘behind the scenes’* experience at the professional AFL club men support. This included alluding to the potential for bumping into players and access to professional footy settings for the program sessions. The program was marketed as an AFL club-specific program	Rural program marketing refers to the program as being *‘footy themed’* in the text and images used, with no mention of specific club affiliation. The phrases *‘no footy experience required’, ‘meeting likeminded men’, ‘having a laugh’, ‘limited places available’* and the potential for *‘mental health benefits’* is highlighted in flyers and social media posts. The fact the program is free of charge was also highlighted	Rural marketing strategies were informed by stakeholder focus groups. Adaptation reasons include to: • make clear there is no affiliation with specific football clubs • make clear that all eligible men are welcome, regardless of their football experience • highlight potential mental health benefits and the opportunity to form connections with other likeminded men • use language that may give a sense that men may be one of the lucky few to participate • clearly highlight that the program is free of charge The goal of this adaptation is to increase the appropriateness of the marketing for rural contexts without club affiliations, and to increase the potential reach and health equity relevance of the program	***Most Relevant Qualitative Themes and Sub-Themes:*** - Engaging Rural Men and Diversity - Program Marketing and Cost - A Common Language - Local Club Rivalries ***Exemplar Stakeholder Quotations:*** *‘…pushing the point that they probably don't need to have been a footy or played footy to participate…[…]…If that can be pushed out that you know, they may not have ever had to do a hand ball to be able to participate. (Site 1, FG1)* *‘So we kind of need to develop that kind of feeling here where people feel lucky to be one of the 60’ (Site 2, FG1)* *‘…they formed those social groups at the end and people that maybe had been feeling a bit lonely. Particularly after this lovely year we've just had, I think that could be a real drawcard as well, around coming in, meeting new people. As opposed to perhaps going just purely for the fitness or the health angle.’ (FG2, Site 2, Female)*	This adaptation does not impact intervention delivery or likely effectiveness and is classified as fidelity consistent How effective the adapted marketing strategies for rural areas are unknown and requires evaluation alongside the rural recruitment strategy ***Potential Generalisability:*** The rural marketing strategy could be generalisable for the marketing of Aussie-FIT across rural areas. Although the marketing is specific to the Aussie-FIT program (e.g., *‘footy themed’*), aspects of the approach could be generalisable to other initiatives taking a gender-tailored approach to engaging men or for programs looking to appeal to individuals from diverse backgrounds
Tailoring and loosening the structure of the coach recruitment strategy and coach training mode. This modification is to the context (setting/format and personal) and the training of the implementors (implementer level)	The intention was for coaches to be recruited directly from AFL clubs, but ultimately, three coaches were recruited via the clubs and an additional three coaches were identified by the research team. The coaches attended four-half day program training workshops	Coach role information will be circulated amongst local stakeholders for dissemination via email and/or social media. Stakeholders will be encouraged to share the role information to local football and other sporting clubs, and other community or health organisations. Key characteristics of coaches (e.g., interest in Australian Football; coaching experience, communication skills) to deliver the program are unchanged. Coaches with or without specific club affiliations are eligible. Flexibility of coach training delivery mode will be considered (e.g., partially online) if coordinating face-to-face training is challenging. The content of the training is unchanged	The flexibility with the coach training mode is informed by pragmatism. The coach recruitment strategy is informed by the stakeholder focus groups and the fact that the rural program is not affiliated with AFL clubs. Stakeholders reported that rural football club volunteers were overburdened and, in many cases, unlikely to be able to have any active involvement in Aussie-FIT. Hence, coach recruitment efforts were not targeted solely at local football clubs. The goal of this adaptation is to increase the adoption of the program	***Most Relevant Qualitative Themes and Sub-Themes:*** - A Smaller Fishpond - Trust, Recognition and Credibility: the importance of local champions - Rural Partnerships and Sustainability ***Exemplar Stakeholder Quotations:*** *‘So it would restrict yourself, if you're looking for coaches I wouldn't restrict yourself to the two [site 1] teams by a long shot.’ (FG1, Site1, Male)* *‘…people are over worked in footy clubs now. Like there's a small amount of volunteers doing a lot of the pulling power. So it's yeah. It's, to ask them to do more is probably unrealistic at this point I reckon.’ (FG1, Site1, Male)* *‘So I think one of your key things will be finding absolutely right person on the ground to be your go to person’ (FG3, Site3, Female)*	All coaches bring their own style of delivery, and some may deliver core program components with a higher degree of fidelity than others, which could in turn influence participant health and health behaviour outcomes. However, the adaptation to the coach recruitment strategy and flexibility with training mode itself is unlikely to impact intervention delivery or program effectiveness and is classified as ‘fidelity-consistent’ How effective the coach recruitment strategy for rural areas is unknown and requires evaluation ***Potential Generalisability:*** The adapted coach recruitment strategy and coach training mode could be generalisable to the recruitment of Aussie-FIT coaches across other rural areas and could be applicable to other rural health initiatives
Program partnership organisation modifications for context (organisational level)	Professional AFL clubs were partners in the pilot Metropolitan Aussie-FIT program. Their primary role was to help promote the program to potential participants and to provide a program delivery venue	Partnerships with the West Australian Football Commission, local government authorities, and other community or health organisations will be sought in the rural Aussie-FIT program. Their role will include helping to promote the program, support the identification and recruitment of coaches, and to help identify and liaise with organisations around venue bookings. Stakeholders will be consulted with on an ongoing basis, during and post-program implementation, in an advisory capacity	The rural program partnerships were informed by the stakeholder focus groups and the fact that the rural program is not affiliated with AFL clubs. The goal of this adaptation is to increase the potential sustainability of the program, as well as the program reach, adoption and the acceptability and appropriateness of the implementation effort in rural contexts	***Most Relevant Qualitative Themes and Sub-Themes:*** - Trust, Recognition and Credibility: the importance of local champions - Rural Partnerships and Sustainability ***Exemplar Stakeholder Quotations: ‘****They roll out all AFL down here to the kids and everything. Yeah. So I would probably say they're the best ones to get involved in rolling this program out. (FG1, Site3, Male)* *That's the big word, relationship. For it to be sustainable, you've got to build on it (Male)…. And while we've got budget, we may as well start doing that and work with them.’ (FG2, Site3, Female)*	This adaptation does not impact intervention delivery or likely effectiveness and is classified as fidelity consistent. The role that program partnership organisations have undertaken during rural implementation will be reviewed and discussed with stakeholders following initial implementation efforts, with a view to working towards a sustainable program delivery model ***Potential Generalisability:*** Program partnership organisations either have equivalent organisations across rural areas, or their organisation and employee structure spans across rural Western Australia. Thus, partnerships adopted for rural implementation could be generalisable for Aussie-FIT implementation across rural areas and could be applicable to other rural health initiatives
**Program Adaptation**	**Pilot/Metropolitan Aussie-FIT**	**Rural Aussie-FIT**	**Rationale for Adaptation or Rural Specific Considerations**	**Exemplar Quotes and Linked Qualitative Themes Informing Adaptations**	**Core Program Components, Fidelity, and Potential Generalisability**
Tailoring/ tweaking, changes in packaging and materials, and substituting aspects of the existing program to reflect the modified Australian football theme/framing and related program content. These modifications cut across community coach, target group, and organisational levels	Delivered in association with specific professional AFL clubs to specifically engage men that support those teams. *‘Behind the scenes’* program feel, with coaches wearing club shirts and men participating getting a free club/team shirt to wear during the program. Session 1: ‘Behind the scenes’ tour of club facilities, walk around the oval with club insider stories. Session 6: A club player, former player, coach, or other’celebrity’ is invited to the session to talk to the men about the football club and reflect on setbacks they have overcome in their career, and how men might relate to setbacks they may experience	An Australian Football themed program, not delivered in association with any specific local or non-local clubs, to appeal to men that may have an interest in Australian Football. Local community program, run by local coaches, with coaches wearing Aussie-FIT shirts, and participants wearing an Aussie-FIT team t-shirt. Session 1: Will likely differ across rural programs depending on the context, but often a walk around the oval, and sharing stories about the history of football in the area or local clubs. Session 6: If a local football guest is not available to come and speak to the men, the coach and participants will share stories of when they have experienced a setback (football related or other)	The goal of these adaptations was to improve intervention fit for the context. The reason for these adaptations was the location/accessibility. Rural contexts with no access to professional AFL club facilities. Program is not affiliated with professional AFL clubs. The program framing and theme were informed by stakeholder focus groups. Stakeholders did not discuss specific program content, with these adaptations made by the research team. Session 1: Rural context with no access to professional AFL club facilities. Session 6: Access to a ‘celebrity guest’ likely more difficult in rural contexts, without AFL club affiliation. Where relevant, program materials (e.g., participant booklets) were tweaked to correspond with these adaptations (e.g., removing or replacing mentions of ‘your club’)	***Most Relevant Qualitative Themes and Sub-Themes:*** - A Common Language - Popularity of Australian Football - Local Club Rivalries ***Exemplar Stakeholder Quotations:*** *‘…you're on a winner with the access to you know, something that is common language which is AFL. Everyone, every dad, and their kids… male has had exposure to it. May have loved it or hated it, but at least they're aware of it. And it's in your face every day, in the paper.’ (FG1, Site 2, Male)* *‘…it probably wouldn't be best affiliated to clubs down here. Because yeah, you'll get ones that are just affiliated to that club that will go and then you might not get the others coming…’ (FG1, Site3, Female)*	Core elements or functions of the program are preserved, and this modification is deemed fidelity consistent. The theoretical basis, behaviour change techniques utilised, and the program content designed to support men to make positive changes to their health behaviours have not been adapted. No tour around professional football settings and potential lack of a ‘celebrity’ appearance links to the rural program not being associated to professional clubs. This removes an aspect of the original program ‘hook’, which could influence how attractive or engaging the program is for some men ***Potential Generalisability:*** These adaptations are likely generalisable to many Western Australian and other rural contexts. For generalisability to parts of Australia where Australian Football is less popular, modifying the sport of choice for the program theme may be a consideration. Using sport as a program ‘hook’ could be applicable to other rural health initiatives to engage participants
Contextual modifications made to the program delivery setting and time of year the program is delivered. These modifications were made for the target group	Professional AFL club settings often with access to a gym onsite. The time of year that programs were delivered varied, with recruitment during the early months of the football season a deliberate consideration. If men were not available for the scheduled program, then they were not enrolled in the program	Local accessible rural amateur Australian Football settings. Program timings are likely to vary across the three rural sites. The peak winter months should be avoided in sites 2 and 3, and the farming season avoided in site 1	The goal of these adaptations is to improve intervention fit for the context. The reason for these adaptations was the location, accessibility, and social context (local climate and employment). Rural context with no access to professional football club facilities. The program venue choices and delivery timings were informed by pragmatism and considerations raised in stakeholder focus groups. In site 1, there was only one possible football oval in town. In sites 2 and 3 where there was more than one potential option, the venue was selected based on availability, accessibility, and potential to reach men from diverse backgrounds. In site 1, the program was not scheduled during farming season in site 1, and not scheduled during winter months in sites 2 and 3	***Most Relevant Qualitative Themes and Sub-Themes:*** - Engaging Rural Men and Diversity - Accessibility and Rural Football Settings - Seasonality, Work and Weather ***Exemplar Stakeholder Quotations:*** *‘Public transport, there is no public transport….[…..]… So central, wherever the programs being run.’ (FG1, Site 2, Male)* *‘…location-wise and that's where [venue name] would be a good one, because that's the area where there is, a lower socioeconomic area, state housing, some Aboriginal involvement there.’ (FG3, Site 3, Male)* *‘…weather is a bit of an issue down here. So if you're having an outdoor program and you're doing it in July, you're not going to get many participants…’ (FG1, Site2, Female)* *‘…every eligible bloke disappears over harvest time.’ (FG2, Site 1, Female)*	These modifications do not impact core elements or functions of the program and are deemed fidelity consistent ***Potential Generalisability:*** Local contextual factors (e.g., weather, employment) will vary across rural sites in Australia which will influence the most appropriate program delivery settings and time of year to deliver Aussie-FIT or other health initiatives to engage the target population

**Relevant Questions from the FRAME-IS and FRAME addressed within this table, as appropriate, are:**

Q1 – Briefly describe the implementation strategy and modifications; Q2 – What is modified?; Q3—What is the nature of the content, evaluation, or training modification?

Q4 – Potential relationship to fidelity/core elements? Q5—What is the goal and reasons for modification? Q6—What is the level of the rationale for modification? Q7—At what level of delivery is the modification made (for whom/what is the modification made)?

**Implementation Terminology**

Implementation strategies – activities and techniques that aim to support the adoption, implementation, and integration of an intervention into practice

Adoption – how many providers deliver an intervention

Fidelity – delivery of an intervention as intended

Reach—how many and the representativeness of participants that receive an intervention

The potential generalisability of the adaptations for Aussie-FIT implementation in other rural Australian contexts beyond the three sites and to other health initiatives or target populations within rural Australia are also considered

### Focus group findings

#### Limited appeal of existing services to men

Access to existing physical activity and weight management services varied by site. Stakeholders in site 1 reported a *“lack of choice”* and suggested that this was likely to be the case in similarly small towns across rural Australia. The one gym in site 1 was expensive, posing a significant barrier to accessing the facility for many community members *‘watching their money’.*

*”rural, regional, remote is a different ball game as well. Just options are so much less out here compared to the city...”* (Site 1, FG1, Male)

When opportunities to participate in mixed-gender programs were presented, few men were reported to engage. One stakeholder involved in delivering some of the few local fitness classes, alluded to a*”stigma”* associated with the opportunities available, suggesting they were viewed as more of a *“feminine type”* of program, or *“not tough enough”* and at odds with many men’s masculine identities.

*“…running the fitness classes and stuff at work. There are no men. Like in the classes, there's none. And I think a lot of that is because there is some stigma around like group exercise. I think men, it's like not necessarily a sign of like weakness. It's maybe like a more of like a feminine type thing”* (Site 1, FG1, Male)

Stakeholders in the two larger rural sites, described more options for physical activity. However, in site 2, program options that appealed specifically to men appeared to be limited.

*“…those programs attract a lot of females. More so than the male demographic. You know, the Zumba and they've got you know, all these sorts of things. But it's getting that... the male activity in there, which is lacking”* (Site 2, FG1, Female)

A soccer-based men’s weight loss initiative was running in site 3. This was viewed as appealing to some men, but not others, with an Australian Football theme considered more likely to attract a largely different demographic of local men. One stakeholder discussed how an Australian Football ‘hook’ would appeal to him, but that he (and others with similar sporting interests) would be averse to participating in a soccer-based program.

*“… most of them were soccer players, because at end of the day, if you're not…. I was a footballer, so a footballer, doesn't go and play soccer. You just go, ‘nope, they're different!’. And that's how it is.”* (Site 3, FG3, Male)

### A common language

#### Popularity of Australian Football

Stakeholders depicted Australian Football as being a connection point for social interaction amongst peers for (many) men in Western Australia, particularly in *“footy-mad”* rural towns.

*“I can understand the appeal, AFL being the common language of WA [Western Australian] males. Yeah, it's interesting when I get some of my male colleagues together it's you know, how dogs normally greet each other? Well, they talk football. You know, just to work out where they fit in it. I always sit back and go ‘yeah okay, that's fascinating.”* (Site 2, FG1, Male)

After the Aussie-FIT pilot study presentation, stakeholders reflected on the program’s popularity when delivered in association with professional AFL clubs in metropolitan Perth, the pride the men showed in participating, and the obvious “*adulation”* for their club. One stakeholder referred to the AFL club links and guest appearances from current or former players, as being a valuable form of “*currency*” that would be attractive to footy-oriented prospective participants. However, the need for a different approach in rural towns without access to professional football settings was recognised.

*“I can see why you had such a popular uptake in Perth. Based, you know, you've got the two AFL sides, bang. You know, and access to the change rooms. Okay, it's all there, packaged nicely. Here it's going to be a little bit harder and you're going to have to look at other alternatives.”* (Site 2, FG1, Male)

#### Local club affiliations and footy exposure

One approach mooted was for Aussie-FIT to be affiliated with local amateur clubs, including using their team colours and football venue for program delivery. However, some stressed that close affiliations with any specific local clubs could lead to the program hitting “*a few snags”*. For example, eligible men with links to other local clubs could be hesitant to take part: *“you would only alienate them”.* Indeed, this stakeholder emphasised how little love is lost between some rival clubs:

*“…. there's that much animosity between the clubs, is a bit like over in Europe with the soccer teams, it's alive and kicking with our footy as well.”* (Site 3, FG3, Male)

Thus, if programs were delivered in association with specific local clubs some stakeholders considered that, in the interest of equity and maximising intervention reach, the program should be delivered *“with each club”*. Whereas others highlighted that close affiliation with any local club could also create barriers to participation for men with limited football experience. Men perceiving others as more skilled, experienced, or active within local football communities, could be “*uncomfortable”* or “*embarrassed”* about joining the program as an inexperienced outsider. Thus, stakeholders universally agreed that program marketing materials should highlight that all eligible men are welcome regardless of footballing experience or skill, and as one stakeholder put it; *“… you don’t have to be skilled in X, Y and Z [to participate].”*

### A smaller fishpond

#### Trust, recognition and credibility: the importance of local champions

Stakeholders agreed that, in rural contexts, getting the right community champions involved would go a long way to ensuring program success. One stakeholder proposed that one well-known local contact would *“give you 30 people”* through their community connections alone. Prospective Aussie-FIT coaches, given their direct involvement in program delivery, were seen as ideally placed to play a critical role in championing the program, given their local knowledge and connections.

*“They'd all have guys who they could tap on the shoulder and say ‘hey, come and join in’. Yeah, I think that would definitely be a good way to say lock in your core staff or people who are going to run the program locally. And then again it's their good reputation in the community that would then potentially attract people. To know that it's not going to be just some gimmick program or something that's not going to have value.”* (Site 3, FG1, Female)

Having trusted and potentially well-known community champions to support program implementation in rural towns was considered important in garnering local trust and attracting men.

*“…there's probably certainly people, [site 2] being a small community, that are very prominent in their community. You know, both for football and the professional lives that they have. So, but yeah, I mean maybe you could leverage their celebrity status. It's completely the wrong word to use, but the recognition they have in the community and the trust that people... and credibility that they hold as well.”* (Site 2, FG2, Female)

Another suggestion was that prospective participants registering their interest may themselves be an ideal and trusted source of participants in rural communities; “*bring a friend or two is probably going to have to be a realistic approach.”*

#### A double-edged sword

Stakeholders indicated that any discomfort around attending a local-club affiliated program could be amplified within close-knit rural communities, where men may know other participants that are more active in the local football community.

*“…people who would then feel uncomfortable about like coming to the program if it's closely aligned with the local team. If they've never really experienced football, but they've always wanted to. And then they think oh but I, I'm not at that level, I'll be embarrassed, I'll you know, the other guys they're all so experienced and I don't feel comfortable. I just wonder is that a risk too, that people may not want to because they think ‘oh I work with him and he's [Club Name] you know, I don't want to look silly…”* (Site 1, FG1, Female)

To maximise the prospect of engaging men from diverse backgrounds within the *“smaller [rural] fishpond”*, stakeholders recommended adopting a multi-faceted recruitment strategy including Facebook promotion, local media sources, and word-of-mouth recruitment through local clubs, organisations, and community champions. Indeed, the power of word of mouth was frequently alluded to as being a particularly important consideration in rural communities. Whilst having the potential to be a key avenue for raising interest in new health initiatives, this was presented as a double-edged sword, with word likely to quickly spread should anyone get *“shitty”*.

*“…with smaller towns and maybe you don't get this in your metro setting. Is... and it can work wonders and it can be really positive. And you know, word of mouth can be positive. But also if something goes awry or if someone gets shitty about something, that goes through [site 1]'s Chinese whispers channels, like nobody's business. So I think it would be a matter of getting like that champion, community champion to stay.”* (Site 1, FG2, Female)

Stakeholders speculated that in urban areas, men would likely be able to limit knowledge of their participation to themselves or to close family or friends should they wish to. This degree of privacy was seen as unlikely to be an option in rural communities, where *“people talk”*. Stakeholders believed that local men will be aware of how their participation is viewed more widely in the community, including any potential threat to their identity as a local man. Given the football program theme, with careful consideration of how the program is marketed, Aussie-FIT was considered well placed to minimise this threat.

*“…All country towns, like people talk. And it's like ‘oh you know, such and such is doing the ol weight loss class’. You know what I mean? So I think guys won't potentially engage because of a fear of that. Whereas like ‘oh you know that like, that footy program. Like you know, the old fellas footy program’. Just you know, something like that is, will make it more likely that they, they buy in. Hence why I think Aussie-FIT will make it. But how you market that will obviously, that'll be the hardest thing...”* (Site 1, FG1, Male)

### Engaging rural men and diversity

#### Program marketing and cost

Whilst the football program hook was deemed fundamental to Aussie-FIT’s potential success in rural towns, the football theme was described as *“a means to an end”.* That is, stakeholders believed that the positive physical health outcomes of participating should be emphasised; “*you need to market the end”*. Particularly in the context of the Covid-19 pandemic, potential mental health benefits linked to participating in the program and meeting like-minded men were also regarded as important to include within promotional materials.

*“…they formed those social groups at the end and people that maybe had been feeling a bit lonely. Particularly after this lovely year we've just had, I think that could be a real drawcard as well, around coming in, meeting new people. As opposed to perhaps going just purely for the fitness or the health angle.”* (Site 2, FG2, Female)

The cost of participating in physical activity programs was regarded as a major barrier to engagement, so ensuring that marketing materials clearly highlight that there is no participation cost was considered important.

*“…you've already ticked a big box by saying it's free. That's the biggest drawcard or a barrier that gets put up is cost.”* (Site 2, FG1, Female)

#### Location of rural football settings

The number of football venues that could host Aussie-FIT varied by site. In site 1 there was only one venue option shared by two local football clubs, with this likely to be the case in similar smaller rural towns. Stakeholders described a lack of public transport across all sites, with those in site 2 specifically recommending the central football precinct to optimise accessibility. In site 3, two main venue possibilities were discussed. The first was the *“premier football facility”* where *“people want to play”,* which was a favoured site should there be availability at this in-demand facility. The second option, with greater availability, was considered well placed for accessibility and promotion to men from diverse socioeconomic backgrounds.

*“…location-wise that's where (venue option 2) would be a good one, because that's the area where there is, a lower socioeconomic area, state housing, some Aboriginal involvement there. So that'd be a good thing”* (Site 3, FG3, Male)

#### Aboriginal engagement

Sports sector specialists in site 3 discussed how even when programs are free to participate in, men from the Aboriginal community would often still not participate. In the context of Australian Football, stakeholders indicated that there is often little involvement of Aboriginal men at local clubs beyond their playing years due to *“entrenched”* barriers, *“little hoops you have to jump through”* and a sense of not being welcome.

*“…even making things free seems as though that the lowest socioeconomic still don't actually get involved all the time. It's that like there's a barrier. And we've seen that with Aboriginal participation….[…]… Aboriginal guys in particular, the barrier is always is that the sport is what actually keeps them involved because they actually feel welcome. And part of it, when they can actually go there and play, they don't necessarily always feel that they're welcome once they're finished.”* (Site 3, FG3, Male)

Aboriginal health specialists also reflected on challenges to engaging Aboriginal men in health initiatives due to issues with trust and deep-rooted barriers to participation. Suggestions for mitigating these barriers were focused on helping to support men to feel more *“culturally comfortable”*, including by seeking *“early buy in [from the Aboriginal community]”*, employing an *“Aboriginal staff member”* or providing bespoke deliveries (*“run it Aboriginal specific”).* Some optimism was expressed that a male-specific Australian football-themed program could potentially have greater appeal than existing weight management services for some Aboriginal men:

*“…not quite comfortable with [mixed-gender program] that would potentially prefer to be in a predominantly non-Aboriginal setting with other men, than an Aboriginal setting with a bunch of women.”* (Site 1, FG2, Female)

#### Seasonality, work and weather

The optimal time of year to engage local men varied across rural sites. In site 1, where a large proportion of the workforce are farmers, scheduling the program to run outside of seeding and harvest months (when *“every eligible bloke disappears”*) was considered essential. In site 2 avoiding the cold, wet and dark winter months was considered the most important factor. Stakeholders in site 3 also advised against scheduling the program in the winter months and highlighted that the high proportion of fly-in-fly-out/rotation workers in the area was an unavoidable barrier to participation. These workers would struggle to participate in structured initiatives requiring consistent (weekly) attendance.

### Rural partnerships and sustainability

The concept of using Australian Football to help engage men was well received by stakeholders, who could see real potential for the program in rural towns.

*“…it's got the potential to be so successful in [site 1] I think. I think it would be... it would be awesome. It would be really, really good.”* (Site 1, FG2, Female)

Whilst this stakeholder’s optimism is palpable, the emphasis on the word “*potential*” in the context of a discussion around program sustainability, hints at prior challenges to health program implementation. Indeed, many stakeholders reflected on programs which had come and gone from their communities due to funding limitations. Moreover, stakeholders cautioned that, particularly where physical activity options were lacking (e.g., Site 1), participants would inevitably be enquiring as to *“what [is] next?”.*

*“Especially with funded programs like that, that run for a very short period of time. It gets very tricky. Because you know, we sort of... we manage, in the network we manage a few funded programs which runs for a year. And there's... people love it and then funding stops. And so yeah, you're going to have definitely that's going to be a challenge and people... participants will definitely ask that question. What's going to happen after 12 weeks? I mean, you do... you're going to do a follow up in three months, but then what next?”* (Site 1, FG2, Female)

Various organisations that could play a role in supporting program implementation were proposed, including those in health, sporting, and community sectors. With a view to sustainability, developing relationships with key local partner organisations was considered vital.

*“That's the big word, relationship. For it to be sustainable, you've got to build on it.”* (Site 3, FG2, Male)

Stakeholders across the sites suggested that the Football Commission was an important potential partner to engage, ideally to help coordinate some local program logistics.

*“… getting the footy commission side of it, so you'd often get the development officers down here conducting it. And that's not saying that they would be the ones taking the sessions, because you can put it out to the local coaches around here who are AFL coaches at any level, to be the ones that administering it. Like WA Footy Commission is the face of it, but I think you'll get better buy in down here type thing.”* (Site 3, FG1, Male)

Rather than being promoted as a metropolitan-based university or professional football club affiliated program (as was the case in Aussie-FIT pilot deliveries), having a well-known and respected football organisation involved in grassroots work as *“the face of it [the program]”* was proposed as a potentially valuable strategy for garnering local community support. This points to the prestige, respect, and potential leverage of this organisation and their employees within their respective regions. Some stakeholders agreed informally to help *“where they can”*, whereas others pledged their support or appeared to take some ownership of the program through their choice of words (e.g., *“that’s when we’ll really yeah, have to drive it…”*).

*“…we'll just give a pledge. [Organisation] are happy to see this you know, delivered within [site 2]. And we'll do what we can to support you in that.”* (Site 2, FG1, Male)

Stakeholders emphasised that rural football clubs are volunteer run, and that capacity for active involvement from club personnel beyond their already stretched capacity was unlikely. Where local government representatives were not present in focus groups, stakeholders highlighted that getting their support would be important, suggesting they would likely *“see the appeal”* and *“be quite receptive”* to Aussie-FIT*.* Where local government representatives were present, they indicated that it may be possible to waive or reduce venue hire fees, and that they would support program promotion efforts. Two important strategies suggested for garnering support from local governments and other organisations were presented. These were: first, to make it *“easy and simple for them to jump on [to support the program]”;* and second, to highlight how supporting the program could help their organisation meet key performance indicators or how it aligns with their broader public health plans.

*“…selling it to them as something that they can tick off the public health plan, and is probably going to be your best bet to get their support”* (Site 3, FG3, Female)

### Aussie-FIT adaptations for rural contexts

Informed by the stakeholder focus group results, adaptations were made to Aussie-FIT and the implementation strategies to be used for implementation in rural contexts (see Table 2). Adaptations were made to program content, participant recruitment strategies, marketing, coach recruitment and training delivery mode, program delivery settings, the football program theme, and program partners.

Focus group findings indicated that suitable Aussie-FIT adaptations (to the program originally delivered in AFL clubs by AFL coaches) for rural implementation include for the program to be delivered in local football settings and adopting an Australian Football theme, without specific affiliation to any local or non-local clubs. Rather than AFL club social media posts, the rural recruitment strategy will include promotion via local media, trusted community sources, local social media pages and word of mouth. The wording of marketing materials will aim to be inclusive of all eligible men. The aim for inclusive language includes emphasising that the program is free and that no prior football experience or skill-level is required. The research team will aim to partner with local trusted football, sporting, Aboriginal-specific and other health organisations, as well as local government authorities to help support inclusivity in program implementation.

Specific adaptations were made to sessions that originally included a stadium tour and Australian Football guest speaker, introducing more flexibility for the delivery of these program components to improve the intervention fit for rural contexts. Core program elements of the original Aussie-FIT program are retained. Namely, the number and length of sessions (i.e., twelve; 90min), mix of education and physical activity components in each weekly session, session topics (e.g., food labels and alcohol), integration of behaviour change techniques, theoretical underpinning (i.e., Self-Determination Theory), fostering of group camaraderie and positive banter, and an overarching Australian Football program theme [18]. The underlying mechanisms of action to support health behaviour changes are unchanged, and thus adaptations made can be considered fidelity consistent. Adaptations to interventions, and strategies to implement interventions, are often necessary to support the fit of the intervention to new contexts, and this can be important to preserve the fidelity of interventions when delivered across different settings [32].

## Discussion

In this study we have explored rural stakeholder perspectives on existing local physical activity and weight management options, and potential barriers and facilitators to implementing an Australian Football-themed men’s health program in rural Western Australia. We describe how focus group findings have informed adaptations to Aussie-FIT program content and implementation strategies for rural deliveries, to understand how to begin to redress rural inequalities. Stakeholder focus groups pointed to a need to and strategies to consider for adapting Aussie-FIT, whilst retaining core intervention elements which are judged essential in the pathway to successful behaviour change.

Findings from the current study suggest that: existing access to weight management services or physical activity initiatives across the rural sites is limited; men are less likely to participate in mixed-gender programs that are available; but gender-tailored place-based approaches could help engage many men in rural areas. Our findings resonate with recent qualitative studies aimed at identifying places that foster well-being among rural men [37] and which have explored masculinities in the context of suicide prevention with rural stakeholders [38]. For instance, Ahmadu et al.(2021) reported on men being open to seeking opportunities for social connection through sporting activities, but also noted that some participants experienced these environments as exclusionary from broader networks or team-related conversations [37]. Stakeholders in the current study cautioned that, although Aussie-FIT would be delivered within rural football settings, the program should not be affiliated with specific local clubs to minimise any incorrect perception that the program is exclusively for men already involved in the local football community. As well as the physical health benefits linked to participation in Aussie-FIT, stakeholders in the current study emphasised that the mental health benefits linked to participation should be highlighted within the marketing materials to help appeal to men who may not be involved in the sporting community and those who may be socially isolated.

Several parallels can also be drawn between our results and a recent qualitative study with local football club representatives exploring barriers and enablers of implementing mental well-being programs within rural Australian football clubs [39]. Hutchesson et al.(2021) reported that program enablers included the social environment offered by rural football club settings, the potential for having a trusted and familiar face from the football community deliver programs, scheduling programs at appropriate times of year, and getting the support of local football and other community organisations [39]. Barriers to program implementation included a lack of volunteers at local football clubs to support the program, cliques within individual clubs and segregation between those involved in different local clubs, and a lack of appropriate community champions to help drive the initiative [39]. Similarly, in the current study, rural stakeholders highlighted the popularity of football in rural communities, the sport’s role in facilitating social connection amongst men, and the important role that trusted local sources can play in supporting the implementation of health initiatives.

‘Fans in training’ programs around the world have typically used the appeal of professional sports clubs, settings, and coaches to engage participants [14, 15, 17, 40]. In the absence of the professional club *‘drawcard’*, replicating some of the inherent recognition and credibility these clubs provide was considered important by stakeholders to help garner local trust. Thus, they recommended delivering the program in association with trusted local organisations and individuals. This aligns with wider literature on engaging men in health interventions, emphasising the importance of using strategies that are congruent with masculine identities and based on trust and rapport [41–44]. Stakeholders characterised those involved in running local clubs (e.g., coaches, committee members) as overburdened volunteers, who would be unlikely to be able to take on a role as an Aussie-FIT coach. Therefore, identifying suitable community champions and organisations to deliver or help champion the program was considered an important challenge to overcome. A recent qualitative study undertaken with stakeholders experienced in sustaining public health programs highlights that finding key champions within local community organisations is an important facilitator to support sustainment, but that over reliance on individual champions was viewed as a potential risk to the longevity of programs [45]. Maintaining communication with stakeholders from key organisations throughout the adaptation and implementation of Aussie-FIT in rural towns, could help to facilitate future decisions around intervention scaling and sustainability.

## Strengths and limitations

Focus groups were undertaken across three rural towns with varied population sizes and demographics. This meant that similarities and differences across sites could be acknowledged within the analysis and reporting, allowing for context specific factors to be considered in the adaptation of Aussie-FIT. The use of established frameworks for reporting content and implementation strategy adaptations allows the reader to easily establish what and why adaptations were made for rural contexts. A diverse range of stakeholders participated in the focus groups within and across rural sites. Women and men participated, including men (*n* = 7 aged 30–65years) within the target age range for the Aussie-FIT program. The range of perspectives, expertise, and local knowledge expressed within the focus groups strengthens this study.

Sites in this study are classified as ‘inner’ or ‘outer’ regional, and travel time to the nearest city from these sites ranges from 1.5 to 5h by car. Thus, some findings from this study may not be generalisable to more rural or remote settings with very low populations. The views of the women (*n* = 11) and younger men (*n* = 5 aged 20–29years) who participated in the focus groups may not represent the Aussie-FIT target group. Moreover, although efforts were made to specifically engage target group men (aged 35-65years with a Body Mass Index > 28), this proved challenging unless these individuals believed that participating in focus groups fell within the remit of their employment (e.g., health promotion or sporting sectors). However, all participants lived and worked in rural communities, had at least some exposure to local health and football settings, and had an understanding of community health behaviours and how masculinity is expressed locally. Due to scheduling issues, one relatively short focus group (Site3, FG1; 39min) had only two participants, both from the same organisation. Although this may have limited the range of expert input provided within this individual focus group, these participants did have highly relevant expertise and experience, and two further focus groups with stakeholders from varied backgrounds were undertaken in this site. Whilst adaptations made to the Aussie-FIT program were based on stakeholder feedback, the authors took a pragmatic approach to what adaptations were feasible within the project’s budget and timelines. For example, one stakeholder’s suggestion, to deliver an Aboriginal specific program, was beyond the scope of this work. Well-funded projects that utilise co-design approaches with meaningful involvement of Aboriginal individuals, organisations and researchers would be required for such work.

## Conclusion

This study supports understandings of the health promotion landscape in rural areas for men, with a focus on barriers and facilitators to engaging men via local Australian Football settings, and the adaptation of a successful metropolitan-based men’s health program for delivery in rural contexts. Rural areas were described as *‘a different ball game’* when compared to urban areas due to limitations with local services and resource. The power of word of mouth in smaller rural communities was highlighted as a double-edged sword, having the potential to influence the implementation of health programs positively or negatively. In the absence of professional club settings, leveraging recognised and credible local community sources to affiliate with, deliver, or otherwise champion the program was viewed as fundamental to the success of the program, both in the short term and for potential future sustainability. Assessing the potential program reach, and implementation barriers and facilitators of the adapted Aussie-FIT program when delivered in rural contexts is now required. These findings have implications for adapting health promotion programs for men in rural areas within non-professional sporting contexts as one means to help redress rural inequalities in men’s health provisions.

## Supplementary Information

**Additional file 1.**

## Data Availability

The data analysed for this paper can be made available upon reasonable request to Matthew David McDonald.

## References

[1] Alston L, Jacobs J, Allender S, Nichols M (2020). A comparison of the modelled impacts on CVD mortality if attainment of public health recommendations was achieved in metropolitan and rural Australia. Public Health Nutr.

[2] Australian Institute of Health and Welfare. Rural & remote health, AIHW, Australian Government 2019. www.aihw.gov.au/getmedia/838d92d0-6d34-4821-b5da-39e4a47a3d80/Rural-remote-health.pdf.aspx?inline=true. Accessed 01 April 2022.

[3] O'Connor A, Wellenius G (2012). Rural–urban disparities in the prevalence of diabetes and coronary heart disease. Public Health.

[4] NCD Risk Factor Collaboration (2019). Rising rural body-mass index is the main driver of the global obesity epidemic in adults. Nature.

[5] Calder R. Obesity rate depends on where you live 2019. www.mitchellinstitute.org.au/news/obesity-rate-depends-on-where-you-live/. Accessed 02 June 2022.

[6] Moreno-Llamas A, García-Mayor J, De la Cruz-Sánchez E (2021). Urban-rural differences in trajectories of physical activity in Europe from 2002 to 2017. Health Place.

[7] Guo Z, Jiang Y, Huffman SK. Agricultural mechanization and BMI for rural workers: a field experiment in China. Iowa State University, Department of Economics (2018). https://core.ac.uk/download/pdf/212816968.pdf. Accessed 02 June 2022.

[8] Pickett W, King N, Lawson J, Dosman JA, Trask C, Brison RJ (2015). Farmers, mechanized work, and links to obesity. Prev Med.

[9] Porter GC, Laumb K, Michaud T, Brito F, Petreca D, Schwieger G (2019). Understanding the impact of rural weight loss interventions: a systematic review and meta-analysis. Obes Rev.

[10] Punt SE, Kurz DL, Befort CA (2020). Recruitment of men into a pragmatic rural primary care weight loss trial. Am J Mens Health.

[11] Australian Government (2019). National Men's Health Strategy 2020–2030, Australia, 2019. www.health.gov.au/sites/default/files/documents/2021/05/national-men-s-health-strategy-2020-2030.pdf. Accessed 02 June 2022.

[12] McDonald MD, Hunt K, Sivaramakrishnan H, Moullin J, Avenell A, Kerr DA (2022). A systematic review examining socioeconomic factors in trials of interventions for men that report weight as an outcome. Obes Rev.

[13] Wyke S, Bunn C, Andersen E, Silva MN, Van Nassau F, McSkimming P (2019). The effect of a programme to improve men’s sedentary time and physical activity: The European Fans in Training (EuroFIT) randomised controlled trial. PLoS Med.

[14] Kwasnicka D, Ntoumanis N, Hunt K, Gray CM, Newton RU, Gucciardi DF (2020). A gender-sensitised weight-loss and healthy living program for men with overweight and obesity in Australian Football League settings (Aussie-FIT): a pilot randomised controlled trial. PLoS Med.

[15] Hunt K, Wyke S, Gray CM, Anderson AS, Brady A, Bunn C (2014). A gender-sensitised weight loss and healthy living programme for overweight and obese men delivered by Scottish Premier League football clubs (FFIT): a pragmatic randomised controlled trial. Lancet.

[16] Maddison R, Hargreaves EA, Jiang Y, Calder AJ, Wyke S, Gray CM (2020). Rugby Fans in Training New Zealand (RUFIT-NZ): protocol for a randomized controlled trial to assess the effectiveness and cost-effectiveness of a healthy lifestyle program for overweight men delivered through professional rugby clubs in New Zealand. Trials.

[17] Petrella RJ, Gill DP, Zou G, De Cruz A, Riggin B, Bartol C (2017). Hockey fans in training: a pilot pragmatic randomized controlled trial. Med Sci Sports Exerc.

[18] Quested E, Kwasnicka D, Thøgersen-Ntoumani C, Gucciardi DF, Kerr DA, Hunt K (2018). Protocol for a gender-sensitised weight loss and healthy living programme for overweight and obese men delivered in Australian football league settings (Aussie-FIT): a feasibility and pilot randomised controlled trial. BMJ Open.

[19] Kwasnicka D, Donnachie C, Thøgersen-Ntoumani C, Hunt K, Gray CM, Ntoumanis N (2021). The Aussie-FIT process evaluation: feasibility and acceptability of a weight loss intervention for men, delivered in Australian Football League settings. Psychol Health.

[20] Hunt KWS, Bunn C, Donnachie C, Reid N, Gray C (2020). Scale up and scale out of a gender-sensitised weight management and healthy lifestyle programme delivered to overweight men via professional sports clubs: the wider implementation of Football Fans in Training (FFIT). Int J Environ Res.

[21] White M, Adams J, Heywood P (2009). How and why do interventions that increase health overall widen inequalities within populations. In:. Soc Inequality Public Health.

[22] Petticrew M, Tugwell P, Kristjansson E, Oliver S, Ueffing E, Welch V (2012). Damned if you do, damned if you don't: subgroup analysis and equity. J Epidemiol Community Health.

[23] Most Popular Sports in Australia 2017. www.topendsports.com/world/lists/popular-sport/countries/australia.htm. Accessed 02 June 2022.

[24] Sustainable Health Review (2019). Sustainable Health Review: Final Report to the Western Australian Government. Department of Health, Western Australia. ww2.health.wa.gov.au/~/media/Files/Corporate/general-documents/Sustainable-Health-Review/Final-report/sustainable-health-review-final-report.pdf. Accessed 02 June 2022.

[25] Chronic Disease Prevention Directorate (2017). Western Australian Health Promotion Strategic Framework, 2017–2021. Department of Health, Western Australia. ww2.health.wa.gov.au/~/media/Files/Corporate/Reports-and-publications/HPSF/WA-Health-Promotion-Strategic-Framework-2017–2021.pdf. Accessed 02 June 2022.

[26] Wiltsey Stirman S, Gamarra JM, Bartlett BA, Calloway A, Gutner CA (2017). Empirical examinations of modifications and adaptations to evidence-based psychotherapies: Methodologies, impact, and future directions. Clin Psychol Sci Pract.

[27] Stirman SW, Gutner CA, Crits-Christoph P, Edmunds J, Evans AC, Beidas RS (2015). Relationships between clinician-level attributes and fidelity-consistent and fidelity-inconsistent modifications to an evidence-based psychotherapy. Implement Sci.

[28] Stirman SW, Baumann AA, Miller CJ (2019). The FRAME: an expanded framework for reporting adaptations and modifications to evidence-based interventions. Implement Sci.

[29] Evans RE, Craig P, Hoddinott P, Littlecott H, Moore L, Murphy S (2019). When and how do ‘effective’ interventions need to be adapted and/or re-evaluated in new contexts? The need for guidance. J Epidemiol Community Health.

[30] Pfadenhauer LM, Gerhardus A, Mozygemba K, Lysdahl KB, Booth A, Hofmann B (2017). Making sense of complexity in context and implementation: the Context and Implementation of Complex Interventions (CICI) framework. Implement Sci.

[31] Escoffery C, Lebow-Skelley E, Haardoerfer R, Boing E, Udelson H, Wood R (2018). A systematic review of adaptations of evidence-based public health interventions globally. Implement Sci.

[32] Moore G, Campbell M, Copeland L, Craig P, Movsisyan A, Hoddinott P (2021). Adapting interventions to new contexts – the ADAPT guidance. BMJ.

[33] The Australian Statistical Geography Standard Remoteness Structure (2016). www.abs.gov.au/websitedbs/D3310114.nsf/home/remoteness+structure. Accessed 02 June 2022.

[34] Australian Institute of Health and Welfare (2022). Rural and remote Australians. www.aihw.gov.au/reports-data/population-groups/rural-remote-australians/links-other-information#:~:text=The%20term%20%27rural%20and%20remote,regional%2C%20Remote%20or%20Very%20remote. Accessed 02 June 2022.

[35] Ritchie J, Spencer L (2002). Qualitative data analysis for applied policy research.

[36] Miller CJ, Barnett ML, Baumann AA, Gutner CA, Wiltsey-Stirman S (2021). The FRAME-IS: a framework for documenting modifications to implementation strategies in healthcare. Imp Sci.

[37] Ahmadu M, Herron RV, Allan JA, Waddell CM (2021). Identifying places that foster mental health and well-being among rural men. Health Place.

[38] Trail K, Oliffe JL, Patel D, Robinson J, King K, Armstrong G (2021). Promoting healthier masculinities as a suicide prevention intervention in a regional Australian community: a qualitative study of stakeholder perspectives. Front Sociol.

[39] Hutchesson H, Dollman J, Baker A, Kernot J (2021). Barriers and enablers to implementing mental well-being programs through Australian rural football clubs—a qualitative descriptive study. Health Promot J Austr.

[40] Maddison R, Hargreaves EA, Wyke S, Gray CM, Hunt K, Heke JI (2019). Rugby Fans in Training New Zealand (RUFIT-NZ): a pilot randomized controlled trial of a healthy lifestyle program for overweight men delivered through professional rugby clubs in New Zealand. BMC Public Health.

[41] McDonald MD, Dombrowski SU, Skinner R, Calveley E, Carroll P, Elders A (2020). Recruiting men from across the socioeconomic spectrum via GP registers and community outreach to a weight management feasibility randomised controlled trial. BMC Med Res Methodol.

[42] Hunt K, Gray CM, Maclean A, Smillie S, Bunn C, Wyke S (2014). Do weight management programmes delivered at professional football clubs attract and engage high risk men? a mixed-methods study. BMC Public Health.

[43] Lefkowich M, Richardson N, Robertson S (2017). “If we want to get men in, then we need to ask men what they want”: pathways to effective health programing for men. Am J Mens Health.

[44] Grace B, Richardson N, Carroll P (2018). “... If you’re not part of the institution you fall by the wayside”: service providers’ perspectives on moving young men from disconnection and isolation to connection and belonging. Am J Mens Health.

[45] Crane M, Nathan N, McKay H, Lee K, Wiggers J, Bauman A (2022). Understanding the sustainment of population health programmes from a whole-of-system approach. Health Res Policy Syst.

