# Supplementary material for: ‘A Different Ball Game’: Adaptation of a men’s health program for implementation in rural Australia
Source: BMC Public Health. 2023 Jul 19;23:1387. doi: 10.1186/s12889-023-16247-w (PMC10355060; doi:10.1186/s12889-023-16247-w)
Supplement: Supplementary file 1 — Additional file 1. [file 12889_2023_16247_MOESM1_ESM.docx]

**Appendix 1: Interview Topic Guide – Stakeholders**

**NOTE:** **The following content may be adapted depending on whether the interview is with an individual or a focus group.**

Acknowledgement of country, specific to area the interview is undertaken [for focus groups]

Welcome. The discussion today will last around one hour. We are going to talk about how a program called Aussie-Fans in Training (Aussie-FIT) might be helpful for men living in non-metro, and rural/regional areas of Australia, and we’d like to seek your views on how we can ensure the program is appropriately customised to suit men in your area. Aussie-FIT is a footy-themed, free men’s weight loss program that we have run in Perth. With some funding from the Department of Health we are looking to see if we can now run the Aussie-FIT in in rural and regional WA, including X [name location of interview]. It’s a program for men to help them lose some weight, by helping them become more active and to eat better. An important attraction of the Aussie-FIT program was the link to AFL clubs (Eagles and Dockers) and the ‘footy feel’ of the program. The program involved 12 weekly 90-minute workshops delivered to groups of 15 men by a coach with an AFL background at footy clubs. The program was delivered at footy clubs (East Perth Oval and Freo Training Facilities) and involved the guys doing some footy related training exercises. A key component of the program is the educational components designed to help motivate the men taking part to make some changes to what they eat and how active they are outside of the weekly sessions, and beyond the 12 week program.

The aim of today’s discussion is to help us work out how the Aussie-Fans in Training (Aussie-FIT) program can be run in [area name] to reach a variety of men. We are keen to learn from and work with stakeholders who are from this area, or are experienced in doing similar work, to help us understand the best ways that the program can be designed to be delivered in [area name] and/or other rural/regional areas, and what changes may need to be made to the program to appeal to men from different backgrounds. Anything you say is important to me so please don’t be afraid of speaking your mind. I will audio-record the discussion today. There are no right or wrong answers – just ideas and opinions, which are all valuable to us.

**It will be useful to introduce ourselves to each other, say something about who you are, where you are from, your job or voluntary work, your connection with or how long you have lived in *[area name]* or other rural/regional areas. I will start…**

**Before we speak specifically about the Aussie-FIT program, the initial questions relate to what might make it easy or difficult for people living in [area name], to be active and eat a healthy diet.**

- What do you think makes it difficult to be active in [area name]? What makes it easier to be active? [prompt: park/green spaces, cost of gyms/groups etc., sports clubs, infrastructure for walking/cycling, convenience of car use, lack of options, weather, knowledge]
- Are there any differences in what makes it easy or difficult to be active between men and women in [area name]? [prompt: weight loss groups, sports clubs, societal/cultural expectations]
- What makes it difficult to eat a healthy diet in [area name]? What makes it easier to eat a healthy diet? [prompt: knowledge of what is/isn’t healthy, cost, availability, support from family/friends]
- Are there any differences in what makes it easy or difficult to eat well between men and women in [area name]? [prompt: weight loss groups, societal/cultural expectations]
- Do some of the factors discussed around being active and eating a healthy diet relate generally to more rural areas in Australia or are they mostly specific to [area name]?

**The next questions are about what kind of community health promotion programs are or have been available in [area name] and what general factors may be important to consider for running these types of programs.**

- Can you describe any examples of community health promotion programs (e.g. weight loss or physical activity programs) that are available or have been available in the area? How successful have these programs been? What challenges have you or others faced in running local programs?
- Do you know of any programs that were originally delivered in urban/city areas like Perth, before being implemented in [area name] or other more rural areas? Which programs?
- Are there any key factors/challenges that need to be considered when bringing any program from the city to [area name] or to other more rural areas? [prompt: seasonal work, weather]
- Is there or has there been anything targeted towards men? If you were to set up a group-based face to face health promotion (e.g. physical activity/weight management) group program specifically for men in [area name], what kind of practical factors might you consider? [prompts: time of day, venue, appeal of program, seasonality/weather]
- Is there anything that might encourage or discourage men in [area name] from participating in such programs? [prompts: time of day, program appeal/‘hooks’, seasonality, other commitments/family/work, societal expectations of men]

**An important draw of the Aussie-FIT program was the link to AFL clubs (Eagles and Dockers) and the fact the program was delivered at club facilities in Perth. For example, the program was publicised via the clubs social media/websites, men got behind the scenes tours, visits from a first team player and the men got club t-shirts** **[researcher shows pictures/short video of Aussie-FIT in action on an I-Pad]. These ‘hooks’ were very effective but maintaining these links with AFL clubs may not be possible, so creating alternatives to get the same ‘buy in’ from men in [area name] will be important. We are really interested in whether the program could be extended to areas like here in [area name].**

- It may not be possible, but if it were, would maintaining a link with the Eagles and/or Dockers be appealing to men in [area name]? Why so/why not? Are the Eagles or Dockers more popular in [area name]?
- How popular are local footy clubs in [area name]? Do many people follow a local team or go to games? Are people interested in the local clubs’ results?
- Could Aussie-FIT draw on the appeal of local footy clubs rather than the AFL clubs? Could the ‘behind the scenes tour/bump into players’ attraction be replicated at local club level? If so, how?
- Some more rural clubs have links with WAFL clubs – do you know if any local [area name] clubs have this kind of link (refer to local example, e.g. Claremont Tigers and the Great Southern)? Could Aussie-FIT draw on WAFL club links, and might this be appealing to local men?
- Do you have any other ideas about how a similar ‘buzz’ or level of interest could be created locally if endorsement/publicity through the AFL clubs was not possible?
- Sometimes programs aimed at men struggle to get enough interest. How might we best recruit men to participate in the Aussie-FIT program within [area name]? [Prompts: Local newspaper, social media, word of mouth, organisation or footy club endorsement/involvement]
- Where might the best venue(s) be for delivering the program that would allow for some practical physical activity and educational parts of the session, but could maintain the feel of it being a men’s space or footy environment?

**Most of the men that participated in Aussie-FIT were guys that lived in the Perth metro area. We want to see how best we can run the program** **in more rural areas to reach men from diverse backgrounds.**

- How might we make the program appealing and inclusive to men from across different backgrounds in [area name]? [Prompt: local community program advocates, language, getting the footy ‘hook’ right]
- How might we best get men from different backgrounds interested in participating in the program? [see above prompt]
- Is there anything that has worked well or has not worked well for trying to reach diverse groups of people in programs before? [Prompt: working with local people]

**Next, we will cover a bit more about what other factors might make it difficult to implement Aussie-FIT within [area name] and what factors could help the program be successful and sustainable**

- What challenges could we face when delivering the program in [area name]? [prompts: recruitment, venues, coaches, participant time, conflict between fans of different clubs]
- What factors might help the program be successful? [prompts: buy in from stakeholders, experienced/enthusiastic coaches]
- Are these factors unique to [area name] or are some of these factors likely similar in other more rural areas? Are there additional or different factors that should be considered for other places that are smaller/bigger/more rural/less rural than [area name]?
- A key challenge is the sustainability of programs. How can programs like Aussie-FIT continue to be offered in a sustainable and inclusive way beyond the piloting stage? [note: we want to make the program appealing for all men, including those less well-off, so providing the program free of charge to men is important]
- What kind of things do we need to think about at this stage to work towards a sustainable offering? [prompt: community connections, asset-based approach, funding streams]

**Thank you all for your time. Do you have any final comments that you would like to add before we finish up?**
